# Cost-effectiveness of Novel Treatment Sequences for Transplant-Ineligible Patients With Multiple Myeloma

**DOI:** 10.1001/jamanetworkopen.2021.3497

**Published:** 2021-03-29

**Authors:** Hedwig M. Blommestein, Margreet G. Franken, Chrissy H. Y. van Beurden-Tan, Nicole M. A. Blijlevens, Peter C. Huijgens, Pieter Sonneveld, Carin A. Uyl-de Groot, Sonja Zweegman

**Affiliations:** 1 Erasmus School of Health Policy & Management, Erasmus University Rotterdam, Rotterdam, the Netherlands; 2 Institute of Medical Technology Assessment, Erasmus School of Health Policy & Management, Erasmus University Rotterdam, Rotterdam, the Netherlands; 3 Department of Hematology, Erasmus Medical Center Cancer Institute, Rotterdam, the Netherlands; 4 Department of Hematology, Radboud University Medical Center, Nijmegen, the Netherlands; 5 Department of Hematology, Cancer Center Amsterdam, Amsterdam University Medical Center, Amsterdam, the Netherlands

## Abstract

**Importance:**

Although the number of treatments for elderly patients with non–transplant-eligible (NTE) multiple myeloma (MM) has increased substantially, evidence is lacking on the clinical effectiveness and cost-effectiveness of novel treatment sequences.

**Objective:**

To determine the optimal sequence of treatment for patients with NTE MM from the perspective of the patient, physician, and society.

**Design, Setting, and Participants:**

Using data from a Dutch observational registry, this economic evaluation combined evidence from network meta-analyses in a patient-level simulation model and modeled time-to-event and types of events from a hospital perspective with a lifetime horizon. Data analysis was performed from June 2019 to September 2020.

**Interventions:**

Thirty treatment sequences, including up to 3 lines of therapy, were compared with bortezomib-melphalan-prednisone (VMP)–lenalidomide-dexamethasone (LenDex)–pomalidomide-dexamethasone (PomDex).

**Main Outcomes and Measures:**

The primary outcomes of the model were overall survival (OS), quality-adjusted life-years (QALYs), costs, and cost-effectiveness.

**Results:**

Sequences starting with daratumumab-VMP (second line: carfilzomib-lenalidomide-dexamethasone or elotuzumab-lenalidomide-dexamethasone) or bortezomib-melphalan-prednisone-thalidomide-maintenance bortezomib-thalidomide (VMPT-VT) (second line: daratumumab-lenalidomide-dexamethasone) had the largest expected OS (7.5 years), which is 3.5 additional life-years compared with VMP-LenDex-PomDex. Total costs per patient for these sequences ranged between $786 024 and $1 085 794. The sequence VMPT-VT-carfilzomib-lenalidomide-dexamethasone–panobinostat-bortezomib-dexamethasone had the most favorable cost-effectiveness ratio ($98 585 per life-year gained and $132 707 per QALY gained vs VMP-LenDex-PomDex).

**Conclusions and Relevance:**

These findings suggest that sequences including novel treatments were highly effective, but the cost-effectiveness ratios were above currently accepted willingness-to-pay thresholds. Treating MM with novel agents necessitates either a large increase in budget or a substantial reduction of drug costs by price negotiations, and these findings can support these reimbursement decisions and price negotiations.

## Key Points

**Question:**

What is the optimal sequence of treatments for patients with multiple myeloma from the perspective of the patient, physician, and society?

**Findings:**

This economic evaluation found that sequences starting with daratumumab-bortezomib-melphalan-prednisone (second line: carfilzomib-lenalidomide-dexamethasone or elotuzumab-lenalidomide-dexamethasone) or bortezomib-melphalan-prednisone-thalidomide-maintenance bortezomib-thalidomide (VMPT-VT) (second line: daratumumab-lenalidomide-dexamethasone) had the largest expected overall survival (7.5 years); total costs per patient for these sequences ranged between $786 024 and $1 085 794. The sequence VMPT-VT-carfilzomib-lenalidomide-dexamethasone-panobinostat-bortezomib-dexamethasone had the most favorable cost-effectiveness ratio ($98 585 per life-year gained, and $132 707 per quality-adjusted life-year).

**Meaning:**

These findings can support clinical decision-making and guideline development, reimbursement decisions, and price negotiations.

## Teaser

This economic evaluation assesses the optimal sequence of treatments for transplant-ineligible patients with multiple myeloma from the perspective of the patient, physician, and society.

## Introduction

Multiple myeloma (MM) is the second most common type of blood cancer, with 50 918 patients with new diagnoses annually in Europe^1^ and 32 270 such patients annually in North America.^2^ Like many cancers, it is incurable, and treatment aims at prolonging the time to disease progression to control disease symptoms and increase overall survival (OS). Eventually, the disease will progress (again), and patients, therefore, often receive several lines of treatment.^3,4,5^

In the past 12 years, the number of treatments for patients with MM has increased substantially. Many randomized clinical trials have been conducted adding next-generation proteasome inhibitors (carfilzomib^6^ and ixazomib^7^), monoclonal antibodies (daratumumab^8,9^ and elotuzumab^10^), new immunomodulatory agents (pomaliomide^11^), and a histone deacetylase inhibitor (panobinostat^12^) to melphalan-prednisone or a 2-drug backbone of either lenalidomide-dexamethasone (LenDex) or bortezomib-dexamethasone (BorDex), which were the standard therapies for relapsed MM. In addition to these 3-drug regimens, carfilzomib-dexamethasone (CarDex)^13^ has been compared with BorDex in the relapsed refractory setting. The 3-drug regimens and CarDex resulted in improved progression-free survival (PFS) compared with the 2-drug regimens LenDex and BorDex. Moreover, for CarDex and carfilzomib-lenalidomide-dexamethasone (CarLenDex) an improvement in OS was observed.^14,15^

Although the availability of effective regimens is embraced by both patients and hematologists, it also imposes challenges. First, as the number of treatment options increases, it is vital to know which sequence is most effective and to compare the OS and quality-adjusted life-years (QALYs) of different sequences. Unfortunately, trials investigating treatment sequences are lacking.^16^ Second, to preserve global access to affordable and effective therapies, it becomes increasingly important to investigate their costs and cost-effectiveness. The prices of novel agents are often high, and, in most regimens, either an expensive drug is added to standard therapy or a more expensive drug replaces standard therapy. Furthermore, many of the novel agents are not limited to a prespecified number of cycles but are administered until disease progression, further increasing the costs.^17^ Although cost-effectiveness studies^3,18,19,20,21^ for elderly patients with non–transplant-eligible (NTE) MM have been conducted, they did not investigate treatment sequences or include recently introduced agents. Therefore, we estimated the clinical effects, costs, and cost-effectiveness of treatment sequences, including all currently available novel agents, for NTE MM.

## Methods

This study uses data from the PHAROS registry.^22,23^ Data collection for that registry and the use of those data for the current study were approved by the ethical committee of the Erasmus University Medical Center Rotterdam in the Netherlands, which waived the need for informed consent because the data were deidentified. This study follows the Consolidated Health Economic Evaluation Reporting Standards (CHEERS) reporting guideline.

### Patient-Level Simulation Model

A model is necessary to combine different data sources and extrapolate data to calculate lifetime costs and effects. We adapted our previously developed patient-level simulation model for elderly patients with NTE MM,^3^ which was based on data from a Dutch observational registry (ie, PHAROS registry).^22,23^ The model is a discrete event simulation consisting of objects and events.^24^ Objects are individual patients and are obtained from a real-world Dutch population of patients with MM aged 65 years or older and, therefore, NTE (median age at first-line treatment, 75 years); events were initiation of a new treatment line or death. Regression models per line of treatment (including coefficients for treatment, and, for the first-line treatment, patient and disease characteristics also) were used to estimate the time to event (TTE), which was defined as the time since the start of treatment to an event) (eAppendix in the Supplement). For the first- and second-line treatments, events were either the start of the next line of treatment or death. The type of event (ie, next line of treatment or death) was based on a logistic regression per line of treatment. From the start of third-line treatment, only time to death was modeled. TTE (as a proxy for time to progression) was selected as the outcome measure because the initiation of a new line of treatment or death is associated with changing costs and effects. The model simulates individual patients, each with his or her own patient and disease characteristics. For each patient, costs and effects were estimated for a maximum of 3 treatment lines according to regression models (eAppendix in the Supplement). We modeled TTE, OS (defined as time from the start of first-line treatment to death), QALYs, costs, and cost-effectiveness. Utility values (on a scale of 0 to 1, where 1 denotes perfect health, and 0 denotes death) for patients treated in clinical practice per treatment and per line of treatment are not available in the literature for patients with NTE MM. To obtain QALYs, we used, as in our previous model,^3^ a mean (SD) utility value of 0.76 (0.21).^25^

### Sequential Treatment Strategies

Novel treatment options for newly diagnosed NTE MM and relapsed or refractory MM were identified from 2 systematic literature reviews and network meta-analyses (NMAs) of randomized phase 3 trials.^26,27^ Both NMAs were updated to include the most recent results from the MAIA,^28^ ALCYONE,^29^ and OPTIMISMM trials.^30^ To estimate the effectiveness of novel treatment sequences, the outcomes from the NMAs were combined with the Weibull regression models for lines 1, 2, and 3. Each model included a reference category for treatment (ie, line 1, melphalan-thalidomide; line 2, BorDex; and line 3, LenDex), and the relative effectiveness of the novel treatments (ie, hazard ratios [HRs] for PFS obtained from the NMAs) was used in the regression models for lines 1, 2, and 3 in the patient-level simulation model (see eFigure 1, eFigure 2, and eFigure 3 in the Supplement). The 95% CIs were used to incorporate uncertainty of treatments’ effectiveness. We identified 30 treatment sequences on the basis of the outcomes of the NMAs and clinical relevance (eTable 1 in the Supplement). We assumed that patients would not receive 2 lenalidomide-based (or 2 bortezomib-based) regimens in a treatment sequence. Hence, patients receiving lenalidomide as the first-line treatment will not receive a lenalidomide-based second-line treatment. However, because many second-line treatments include either lenalidomide or bortezomib, the possible treatment sequences are limited for first-line regimens with both lenalidomide and bortezomib (ie, bortezomib-lenalidomide-dexamethasone [VRD]) or with lenalidomide (eg, daratumumab-lenalidomide-dexamethasone [DaraLenDex]).

### Other Model Parameters

#### Treatment Costs

We retrieved dosing schemes and timing of administrations from Dutch clinical guidelines or, if not available, from randomized clinical trials (eTable 2 in the Supplement). We distinguished time periods and different costs per day for treatments with changing dosing schedules, frequencies, or regimen composition. Regimens that were given continuously (ie, until progression) were assumed to be given until the start of the next treatment or until death, whichever occurred first. For treatments with a maximum treatment duration, we used TTE or the maximum duration, whichever was shortest. Unit costs for drugs as of March 2019 were retrieved from a Dutch pharmaceutical price database (ie, including 6% value-added tax) (see eTable 3 and eTable 4 in the Supplement for details). eTable 5 in the Supplement shows detailed drug costs per day and month.

#### Other Costs

Other resource use included hospitalizations, outpatient visits, and laboratory tests. For agents requiring intravenous administration, we assumed a visit to the day ward per administration. Apart from intravenous drug administration, we retrieved average resource use in clinical practice per month by line of treatment from all elderly patients (aged ≥65 years) of the PHAROS registry (eTable 4 in the Supplement). Unit prices for hospitalization days, intravenous administrations, and outpatient visits were obtained from the Dutch costing manual^31^ and a Dutch MM costing study^32^ (eTable 5 in the Supplement).

### Statistical Analysis

For the cost-effectiveness analysis, costs (presented in US dollars; conversion rate as of January 26, 2021, €1 = $1.2143) and effects were calculated for all treatment sequences from a hospital perspective with a lifetime horizon. The sequence bortezomib-melphalan-prednisone (VMP)–LenDex–pomalidomide-dexamethasone (PomDex) was selected as the base case for the cost-effectiveness analysis to obtain the incremental (ie, additional) effects and costs and the incremental cost-effectiveness ratio (ICER) per life-year (LY) and per QALY gained. To account for the uncertainty of the estimates, all results were obtained using probability distributions for input parameters. Input parameters for resource use and unit costs followed gamma distributions, and the utility values followed a beta distribution. The uncertainty regarding the effectiveness of the treatments was incorporated by drawing HRs from the 95% CIs and 95% credible intervals from the NMAs. We obtained mean ICERs from 5000 simulations. Costs and effects in this study were calculated by applying (1) no discount rates and (2) a discount rate of 4% for future costs and 1.5% for future effects as recommended by the Dutch Costing Manual.^31^

We performed regression analysis in Stata MP statistical software version 16.1 (StataCorp), and the model was built in Excel 365 (Microsoft). Data analysis was performed from June 2019 to September 2020.

## Results

### Outcomes by Line

**Figure 1.**
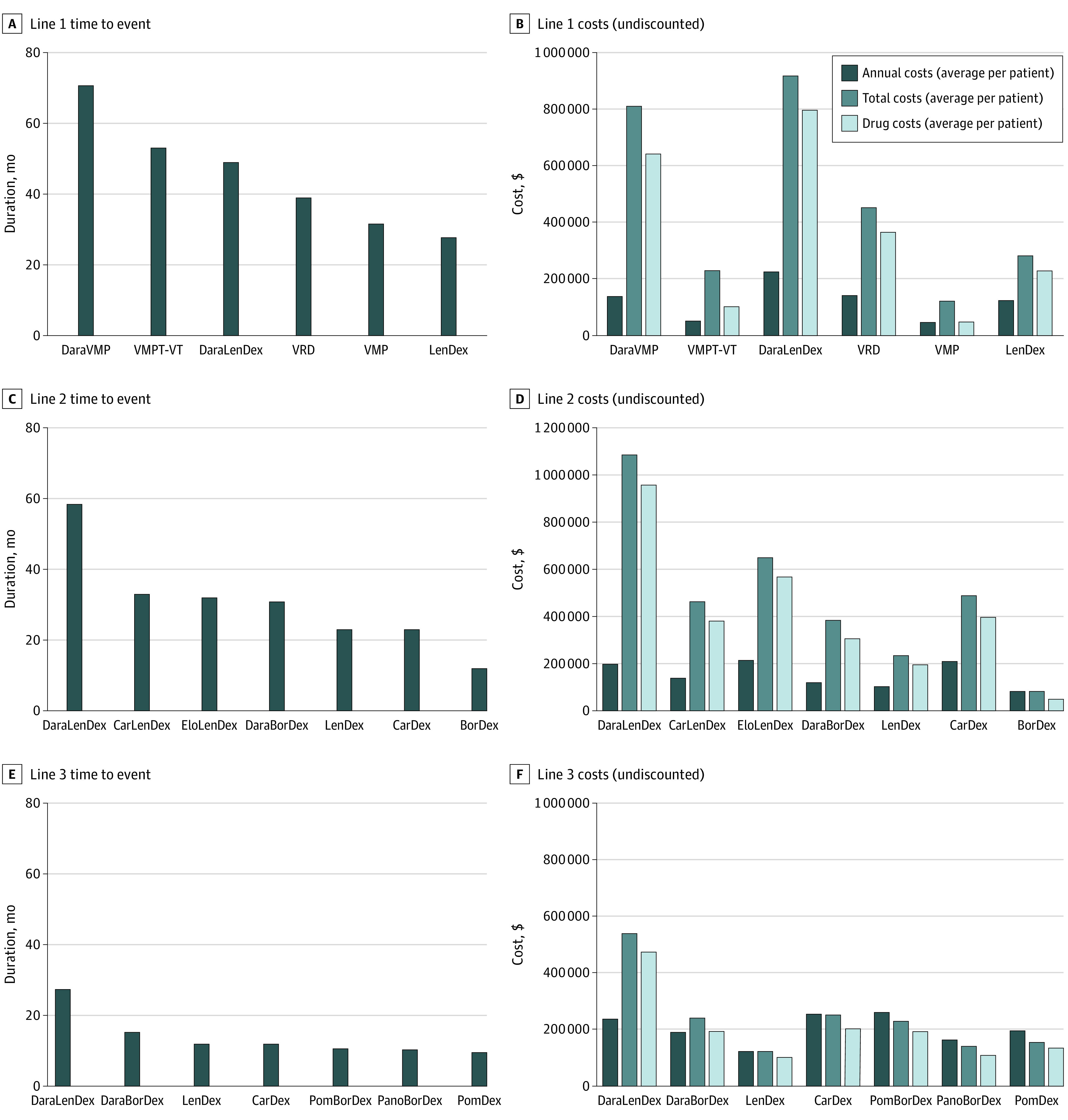
Time to Event and Costs per Regimen for Line 1, 2, and 3 Treatment Sequences BorDex indicates bortezomib-dexamethasone; CarDex, carfilzomib-dexamethasone; CarLenDex, carfilzomib-lenalidomide-dexamethasone; DaraBorDex, daratumumab-bortezomib-dexamethasone; DaraLenDex, daratumumab-lenalidomide-dexamethasone; DaraVMP, daratumumab-bortezomib-melphalan-prednisone; EloLenDex, elotuzumab-lenalidomide-dexamethasone; LenDex, lenalidomide-dexamethasone; PanoBorDex, panobinostat-lenalidomide-dexamethasone; PomBorDex, pomalidomide-bortezomib-dexamethasone; PomDex, pomalidomide-dexamethasone; VMP, bortezomib-melphalan-prednisone; VRD, bortezomib-lenalidomide-dexamethasone; VMPT-VT, bortezomib-melphalan-prednisone-thalidomide-maintenance bortezomib-thalidomide.

Figure 1 shows the undiscounted outcomes stratified by treatment regimen for the first-, second-, and third-line treatments. These figures show the TTE in months and the annual costs, total drug costs, and total costs.

Figure 1A and 1B show that DaraVMP is the most effective first-line regimen (mean [SD] TTE, 71 [24] months vs 53 [19] months for VMPT-VT). DaraLenDex had the highest total, annual, and drug costs. Although TTEs for VMP and LenDex were similar (32 vs 28 months), costs for LenDex were more than doubled compared with those for VMP (total costs, $281 241 vs $119 052; annual costs, $121 618 vs $45 230). The difference in costs is mainly associated with differences in treatment duration; LenDex is given until progression (ie, 28 months), whereas VMP is given with a maximum duration of 9 cycles of 35 days.

In the second and third lines, DaraLenDex had the longest TTE and the highest mean total costs per patient but not the highest annual costs (Figure 1C and 1D and Figure 1E and 1F). Elotuzumab-lenalidomide-dexamethasone (EloLenDex) had the highest annual costs in the second line ($210 302) (Figure 1C and 1D), and pomalidomide-bortezomib-dexamethasone (PomBorDex) had the highest annual costs in the third line ($258 324) (Figure 1E and 1F).

Figure 1C and 1D show that CarLenDex was more effective than CarDex (TTE, 33 vs 23 months). However, total costs in line 2 for CarLenDex (3-drug regimen) were lower than total costs for CarDex (2-drug regimen) ($456 492 vs $481 745). Although CarDex is given until progression (ie, 23 months), carfilzomib is discontinued after 18 cycles of 28 days each in the 3-drug regimen.

### Outcomes for Treatment Sequences

**Figure 2.**
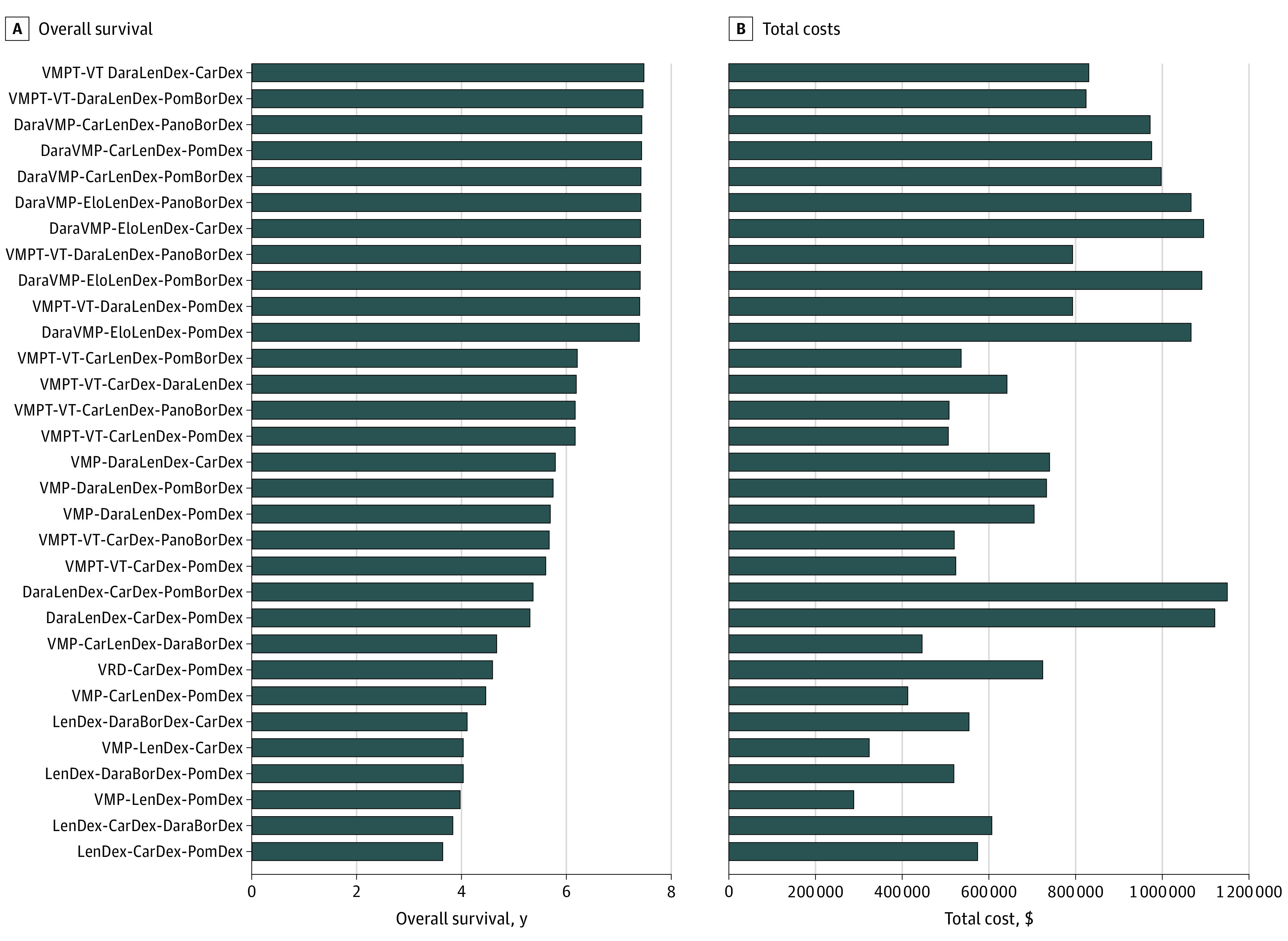
Effects and Total Costs per Treatment Sequence BorDex indicates bortezomib-dexamethasone; CarDex, carfilzomib-dexamethasone; CarLenDex, carfilzomib-lenalidomide-dexamethasone; DaraBorDex, daratumumab-bortezomib-dexamethasone; DaraLenDex, daratumumab-lenalidomide-dexamethasone; DaraVMP, daratumumab-bortezomib-melphalan-prednisone; EloLenDex, elotuzumab-lenalidomide-dexamethasone; LenDex, lenalidomide-dexamethasone; PanoBorDex, panobinostat-lenalidomide-dexamethasone; PomBorDex, pomalidomide-bortezomib-dexamethasone; PomDex, pomalidomide-dexamethasone; VMP, bortezomib-melphalan-prednisone; VRD, bortezomib-lenalidomide-dexamethasone; VMPT-VT, bortezomib-melphalan-prednisone-thalidomide-maintenance bortezomib-thalidomide.

Figure 2 shows the OS and total costs of 31 treatment sequences sorted according to survival. The first 11 sequences with daratumumab as first-line treatment (DaraVMP) or second-line treatment (ie, DaraLenDex) had similar survival outcomes (mean OS, 7.4-7.5 years, which is 3.5 additional life-years compared with VMP-LenDex-PomDex). The costs of these 11 sequences ranged from $786 024 for VMPT-VT-DaraLenDex-panobinostat-lenalidomide-dexamethasone (PanoBorDex) to $1 085 794 for DaraVMP-EloLenDex-CarDex. Compared with our base case VMP-LenDex-PomDex, the additional costs for treating 1 patient with MM may require the health care budget to increase with up to $800 000 per patient. Sequences with first-line treatment with daratumumab yielded higher mean costs compared with sequences with second-line daratumumab treatment. The sequence DaraLenDex-CarDex-PomBorDex had the highest total costs ($1 139 944). Furthermore, although DaraVMP was more effective as a first-line treatment than other first-line treatments such as VMPT-VT, treatment sequences with either DaraVMP or VMPT-VT yielded similar outcomes when DaraLenDex was given as second-line treatment.

Because we assumed that patients would not receive 2 lenalidomide-based regimens in a sequence, the OS of the sequences VRD-CarDex-PomDex (4.6 years), DaraLenDex-CarDex-PomBorDex (5.4 years), and DaraLenDex-CarDex-PomDex (5.3 years) was lower than might have been expected on the basis of their first-line outcomes in Figure 1A and 1B. eTable 6 in the Supplement shows the number of patients with a second and third line (ie, 60%-70% of patients receiving second-line treatments and 35%-50% of patients receiving third-line treatments). eTable 6 in the Supplement also shows detailed discounted and undiscounted effects and costs per treatment sequence. LYs gained from second-line treatments were 3.3 years for sequences with CarLenDex as the second-line treatment and 2.5 years for sequences with LenDex as the second-line treatment; 0% of patients survived up to 20 years.

### ICERs for Treatment Sequence

The Table shows the discounted incremental effects (LYs and QALYs), costs, and the ICERs compared with the base case (VMP-LenDex-PomDex), ranging from $98 585 to $3 762 906 per LY gained. The ICERs for the 11 most effective sequences ranged from $145 643 per LY gained for VMPT-VT-DaraLenDex-PanoBorDex to $232 051 per LY gained for DaraVMP-EloLenDex-PomBorDex. The most favorable sequences, with ICERs ranging from $98 585 to $99 585 per LY gained (ie, $132 707-$134 481 per QALY gained), were VMPT-VT-CarLenDex-PanoBorDex ($110 192 per QALY gained) and VMPT-VT-CarLenDex-PomDex. Apart from these sequences, all ICERs per LY gained were greater than $100 000 compared with VMP-LenDex-PomDex. For patients who cannot receive VMPT-VT as a first-line treatment, DaraVMP-CarLenDex-PanoBorDex, DaraVMP-CarLenDex-PomDex, or DaraVMP-CarLenDex-PomBorDex might be an alternative for these patients from a cost-effectiveness perspective ($195 903-$204 027 per LY gained). The sequences LenDex-CarDex–daratumumab-bortezomib-dexamethasone (DaraBorDex) and LenDex-CarDex-PomDex were dominated by the base case, which means that effectiveness of these sequences was lower, whereas the costs were higher ($568 884 vs $285 619).

**Table.** Outcomes of Treatment Sequences and Incremental Differences Compared With the Reference Case (Discounted)^a^

Treatment sequence	Mean (SD)			Incremental			Costs, $	
	OS, y	QALYs, No.	Total costs, $	LYs gained, No.	QALY gained, No.	Cost, $	Per LY gained	Per QALY gained
VMPT-VT- DaraLenDex-CarDex	7.5 (1.4)	5.6 (1.1)	823 321 (99 619)	3.51	2.61	537 702	153 119	206 016
VMPT-VT- DaraLenDex-PomBorDex	7.5 (1.4)	5.5 (1.1)	816 742 (98 955)	3.49	2.59	531 123	152 366	205 067
DaraVMP-CarLenDex-PanoBorDex	7.4 (1.5)	5.5 (1.2)	963 281 (153 015)	3.46	2.57	677 662	195 903	263 682
DaraVMP-CarLenDex-PomDex	7.4 (1.8)	5.5 (1.2)	967 078 (154 007)	3.46	2.56	681 459	197 143	266 195
DaraVMP-CarLenDex-PomBorDex	7.4 (1.5)	5.5 (1.2)	988 834 (150 514)	3.45	2.56	703 214	204 027	274 693
DaraVMP-EloLenDex-PanoBorDex	7.4 (1.7)	5.5 (1.3)	1 057 083 (157 751)	3.45	2.56	771 464	223 883	301 353
DaraVMP-EloLenDex-CarDex	7.4 (1.6)	5.5 (1.2)	1 085 794 (153 163)	3.44	2.55	800 175	232 496	313 794
VMPT-VT-DaraLenDex-PanoBorDex	7.4 (1.5)	5.5 (1.1)	786 024 (99 565)	3.44	2.55	500 405	145 643	196 237
DaraVMP-EloLenDex-PomBorDex	7.4 (1.6)	5.5 (1.2)	1 081 747 (154 571)	3.43	2.55	796 128	232 051	312 207
VMPT-VT-DaraLenDex-PomDex	7.4 (1.4)	5.5 (1.1)	788 832 (99 696)	3.42	2.54	503 213	147 031	198 115
DaraVMP-EloLenDex-PomDex	7.4 (1.8)	5.5 (1.3)	1 057 142 (157 439)	3.41	2.53	771 523	226 087	304 950
VMPT-VT-CarLenDex-PomBorDex	6.2 (1.5)	4.6 (1.1)	531 163 (51 739)	2.23	1.65	245 544	110 192	148 814
VMPT-VT-CarDex-DaraLenDex	6.2 (1.3)	4.6 (1.1)	635 948 (55 702)	2.21	1.64	350 329	158 759	213 615
VMPT-VT-CarLenDex-PanoBorDex	6.2 (1.3)	4.6 (1.1)	501 931 (48 970)	2.19	1.63	216 312	98 585	132 707
VMPT-VT-CarLenDex-PomDex	6.2 (1.4)	4.6 (1.1)	503 478 (50 730)	2.18	1.62	217 859	99 859	134 481
VMP-DaraLenDex-CarDex	5.8 (1.2)	4.3 (0.8)	733 277 (102 576)	1.81	1.35	447 658	246 757	331 598
VMP-DaraLenDex-PomBorDex	5.7 (0.9)	4.3 (0.8)	726 470 (101 105)	1.77	1.32	440 850	248 717	333 978
VMP-DaraLenDex-PomDex	5.7 (1.1)	4.2 (0.8)	697 907 (103 486)	1.72	1.28	412 288	240 051	322 100
VMPT-VT-CarDex-PanoBorDex	5.7 (1.6)	4.2 (1.1)	515 869 (49 717)	1.70	1.26	230 249	135 507	182 738
VMPT-VT-CarDex-PomDex	5.6 (1.5)	4.2 (1.1)	515 422 (50 189)	1.63	1.21	229 803	140 767	189 920
DaraLenDex-CarDex-PomBorDex	5.4 (1)	4.0 (0.8)	1 139 944 (187 754)	1.39	1.04	854 324	613 518	821 466
DaraLenDex-CarDex-PomDex	5.3 (1)	3.9 (0.8)	1 110 003 (187 209)	1.33	0.99	824 383	618 674	832 711
VMP-CarLenDex-DaraBorDex	4.7 (0.8)	3.5 (0.7)	441 876 (45 743)	0.70	0.52	156 257	223 491	300 495
VRD-CarDex-PomDex	4.6 (1)	3.4 (0.7)	717 756 (102 885	0.62	0.46	432 137	696 994	939 427
VMP-CarLenDex-PomDex	4.5 (0.8)	3.3 (0.6)	409 243 (44 453)	0.49	0.37	123 624	251 866	334 120
LenDex-DaraBorDex-CarDex	4.1 (0.6)	3.0 (0.4)	549 399 (55 010)	0.14	0.10	263 780	1 941 938	2 637 800
VMP-LenDex-CarDex	4.1 (0.8)	3.0 (0.6)	321 034 (36 105)	0.10	0.08	35 415	345 513	442 688
LenDex-DaraBorDex-PomDex	4.0 (0.5)	3.0 (0.4)	514 529 (52 341)	0.06	0.05	228 910	3 762 906	4 578 202
VMP-LenDex-PomDex	4.0 (0.8)	2.9 (0.6)	285 619 (32 401)	Reference case	Reference case	NA	NA	NA
LenDex-CarDex-DaraBorDex	3.8 (0.5)	2.8 (0.3)	601 285 (59 594)	−0.14	−0.10	Dominated	NA	NA
LenDex-CarDex-PomDex	3.6 (0.4)	2.7 (0.3)	568 884 (58 693)	−0.33	−0.24	Dominated	NA	NA

Abbreviations: BorDex, bortezomib-dexamethasone; CarDex, carfilzomib-dexamethasone; CarLenDex, carfilzomib-lenalidomide-dexamethasone; DaraBorDex, daratumumab-bortezomib-dexamethasone; DaraLenDex, daratumumab-lenalidomide-dexamethasone; DaraVMP, daratumumab-melphalan-prednisone-bortezomib; EloLenDex, elotuzumab-lenalidomide-dexamethasone; LenDex, lenalidomide-dexamethasone; LY, life-year; NA, not applicable; PanoBorDex, panobinostat-lenalidomide-dexamethasone; PomDex, pomalidomide-dexamethasone; PomBorDex, pomalidomide-bortezomib-dexamethasone; QALY, quality-adjusted life-year; VMP, melphalan-prednisone-bortezomib; VMPT-VT, melphalan-prednisone-bortezomib-thalidomide-bortezomib-thalidomide; VRD, bortezomib-lenalidomide-dexamethasone.

^a^ Costs are presented in US dollars, conversion rate as of January 26, 2021, €1 = $1.2143.

### Uncertainty Analysis

**Figure 3.**
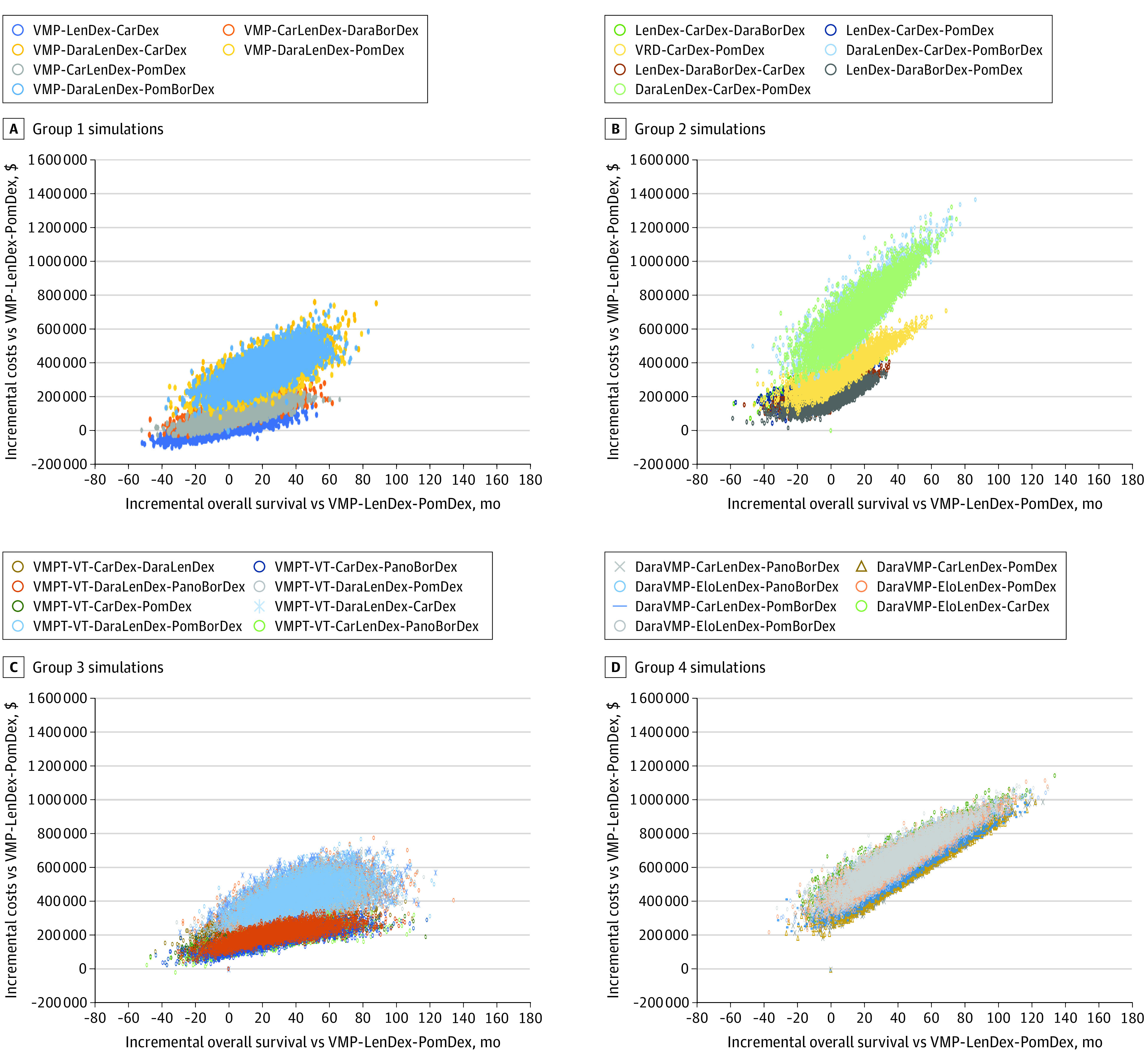
Incremental Overall Survival and Costs Compared With Bortezomib-Melphalan-Prednisone–Lenalidomide-Dexamethasone–Pomalidomide-Dexamethasone (VMP-LenDex-PomDex) BorDex indicates bortezomib-dexamethasone; CarDex, carfilzomib-dexamethasone; CarLenDex, carfilzomib-lenalidomide-dexamethasone; DaraBorDex, daratumumab-bortezomib-dexamethasone; DaraLenDex, daratumumab-lenalidomide-dexamethasone; DaraVMP, daratumumab-bortezomib-melphalan-prednisone; EloLenDex, elotuzumab-lenalidomide-dexamethasone; LenDex, lenalidomide-dexamethasone; PanoBorDex, panobinostat-lenalidomide-dexamethasone; PomBorDex, pomalidomide-bortezomib-dexamethasone; PomDex, pomalidomide-dexamethasone; VMP, bortezomib-melphalan-prednisone; VRD, bortezomib-lenalidomide-dexamethasone; VMPT-VT, bortezomib-melphalan-prednisone-thalidomide-maintenance bortezomib-thalidomide.

Figure 3 shows the incremental OS in months and incremental costs for each of the 5000 simulations per treatment sequence compared with the base case. The individual circles form a cloud, and the spreading of this cloud shows the uncertainty in the outcomes. Figure 3 shows that the 2 dominated sequences, LenDex-CarDex-DaraBorDex and LenDex-CarDex-PomDex, were in more than 50% of the simulations (63% and 54%, respectively) and were more expensive and less effective.

## Discussion

To our knowledge, this study is the first to provide evidence of sequences in terms of clinical effects, costs, and cost-effectiveness in clinical practice for patients with NTE MM. We show the optimal sequences for clinical effects and cost-effectiveness, as well as relative differences. These insights provide valuable evidence additional to data from registration studies and can be used for clinical decision-making, guideline development, reimbursement decisions, and price negotiations.

Although the cost-effectiveness of treatment sequences was not investigated previously, we can compare our results with those of other cost-effectiveness studies. The LYs for patients receiving CarLenDex and LenDex (7.83 and 5.84 years, respectively) of Jakubowiak et al^20^ were much higher than our estimates from second-line treatment with CarLenDex (ie, 3.3 years) and LenDex (ie, 2.5 years). This difference can be explained by the long-term OS predictions from Jakubowiak et al^20^; 20% of their patients treated with CarLenDex survived 20 years and 10% survived 30 years, but 0% of patients in our model survived up to 20 years. Extended follow-up of OS data indicated that the earlier predictions of Jakubowiak et al^20^ were too optimistic because the median OS was 4 years and 65% of patients were not alive at 6 years. Other cost-effectiveness studies are more difficult to compare because of different patient populations (eg, the differences in age of the population^18^ or differences in number of prior treatment lines^33^) and different modeling methods.^18^

Modeling treatment sequences has major advantages. First, it helps to identify the optimal sequence of treatments in terms of effectiveness and to avoid suboptimal decisions. DaraVMP was the most effective first-line treatment, but sequences with this treatment in the first line have limited treatment options for the subsequent lines. For example, the most effective treatment combination in relapsed or refractory MM setting, DaraLenDex, is unlikely to be prescribed after first-line treatment with DaraVMP. As a consequence, the effectiveness of sequences starting with VMPT-VT followed by DaraLenDex yielded similar effects. Sequences with DaraVMP (followed by CarLenDex or EloLenDex as the second-line treatment) and VMPT-VT (followed by DaraLenDex as the second-line treatment) were most effective, with estimated OS up to 7.5 years. This is 3.5 additional LYs compared with VMP-LenDex-PomDex. In addition, we showed that although VRD is more effective than VMP, treatment sequences with VRD as first-line treatment had lower estimated survival. If both bortezomib and lenalidomide are already included in the first-line regime, the possible second- and third-line treatments are currently limited.

In addition to identifying the optimal sequence in terms of effectiveness, our modeling study also provides evidence on costs. For example, total costs of the most effective treatment sequences ranged from $786 024 to $1 085 794 per patient. Compared with our base case VMP-LenDex-PomDex, the additional costs for treating 1 patient with MM may require the health care budget to increase with up to $800 000 per patient.

Furthermore, our results show that sequences with similar effects may greatly differ in health care costs. Although the expected outcomes of the sequences VMP-LenDex-PomDex and LenDex-CarDex-PomDex were similar, the costs of the latter sequence were twice as high ($568 884 vs $285 619). This difference was mainly due to higher costs of LenDex compared with VMP as first-line treatment (total mean costs per patient were $119 052 for VMP and $281 241 for LenDex). In addition, we showed that the most effective sequences did not accrue the highest costs. The sequence DaraLenDex-CarDex-PomBorDex had the highest total costs ($1 139 944) but lower mean OS (5.4 years) compared with the most effective sequences.

Using public list prices, we were able to provide evidence for reimbursement decisions by modeling the ICERs for treatment sequences including novel agents. These ranged from $98 585 to $3 762 906 per LY gained compared with VMP-LenDex-PomDex. Our results show that all ICERs were above currently accepted willingness-to-pay thresholds (eg, up to $96 800 [€80,000] per QALY in the Netherlands^34^ and £30 000 per QALY in the United Kingdom^35^). Sequences with first-line VMPT-VT followed by CarLenDex had the most favorable ICERs ($98 585-$110 192 for the sequences VMPT-VT-CarLenDex-PanoBorDex, VMPT-VT-CarLenDex-PomDex, and VMPT-VT-CarLenDex-PomBorDex). However, VMPT-VT is not commonly used in clinical practice, mainly because of the toxicity of long-term thalidomide administration, thereby hampering its implementation in clinical practice. From a reimbursement perspective, DaraVMP-CarLenDex-PanoBorDex, DaraVMP-CarLenDex-PomDex, or DaraVMP-CarLenDex-PomBorDex might be an alternative for these patients ($195 903-$204 027 per LY gained) because these regimens had the lowest ICERs after the sequences starting with VMPT-VT.

The increased costs for treating MM with the novel agents necessitates either a large increase in budget or a substantial reduction of drug cost imposed by price negotiations. Our results provide crucial information for these negotiations by showing the costs of drugs and health care costs separately, and the model can recalculate the costs and cost-effectiveness for negotiated prices.

### Limitations

This study has limitations that should be addressed. Although modeling treatment sequences provides additional information, assumptions had to be made to make a comparison of treatments that are not head-to-head comparisons. To compare the regimens, we used relative effectiveness between treatment regimens because the relative difference preserves randomization within the trials.^36^ According to the NMA, the HR for PFS was slightly better for VMP compared with LenDex, but with overlapping 95% CIs (VMP vs MPT, HR, 0.83 [95% CI, 0.46-1.51; LenDex vs MPT, HR, 0.94 [95% CI, 0.67-1.30]).^36^ Because the relative effectiveness of DaraVMP compared with VMP was more favorable (HR for PFS in the ALCYONE trial, 0.42)^29^ compared with the relative effectiveness of DaraLenDex compared with LenDex (HR for PFS in the MAIA trial, 0.56),^29^ DaraVMP shows better outcomes in our model compared with DaraLenDex. It should be noted that absolute PFS obtained from comparisons in phase 3 trials are more favorable for continuous LenDex-based regimens (eg, LenDex, 21-39 months; VRD, 43 months; and DaraLenDex, not reached) compared with bortezomib-based regimens (VMP, 17.3-32 months; VMPT-VT, 35 months; and DaraVMP, 36.4 months).^36^ Future research, ideally a head-to-head comparison of DaraVMP and DaraLenDex, should confirm whether DaraVMP is indeed more effective.

We assumed that the HR for PFS would be representative for the HR for TTE to use the NMA outcomes. Although we cannot verify whether this assumption is valid for all treatments, it is supported by the results from the VISTA trial (HR for VMP vs MP for PFS, 0.56 [*P* < .001]; HR for time to next treatment, 0.52 [*P* < .001])^37^ and GIMEMA0305 trial (VMPT-VT vs VMP, HR, 0.58 [*P* < .001] and time to next treatment, HR, 0.52 [*P* < .001])^38^. The number of patients decreases per line of treatment, which is based on the association between TTE and the type of event, as observed in Dutch clinical practice data (ie, PHAROS data). This is, however, comparable to proportions reported for other European countries.^5^ We assumed that the association between TTE and type of event also exists for the novel therapies. Future clinical practice data should confirm this modeled proportion for novel treatments (60%-70% for second-line treatments and 35%-50% for third-line treatments).

In addition, the actual acquisition price may be lower in other countries because of negotiated discounts. Our results (Figure 1) show that drug costs are the major cost factor and illustrate the sensitivity of our results to the drug prices. If the negotiated price reduction has a similar ratio for the drugs, the order of the results will not substantially differ. An advantage of our model is that negotiated discounted prices and prices from other countries can be easily incorporated.

## Conclusions

The findings of this economic evaluation show the relevance of investigating treatment sequences and may help to improve the quality of care for patients with NTE MM and the efficiency of health care delivery. In the context of increasing health care expenditures, evidence on the cost-effectiveness of treatments and on treatment sequences is crucial for ensuring efficient use of limited resources.

## Notes

Supplement. **eAppendix.** Patient Level Simulation Model **eFigure 1.** Forest Plot of NMA Results NTE MM for PFS Line 1 **eFigure 2.** Forest Plot of NMA Results R/R MM for PFS Line 2 **eFigure 3.** Forest Plot of NMA Results R/R MM for PFS Line 3 **eTable 1.** Treatment Sequences Including Relative Effectiveness as Obtained From NMA **eTable 2.** Details of the Treatment Regimens **eTable 3.** Unit Costs of Resource Use **eTable 4.** Costs of Additional Resource Use per Month **eTable 5.** Drug Costs per Regimen per Day and Month Stratified by Treatment Period **eTable 6.** Detailed Discounted and Undiscounted Effects and Costs for Each Treatment Sequence
