# Supplementary material for: Cost-effectiveness of Novel Treatment Sequences for Transplant-Ineligible Patients With Multiple Myeloma
Source: JAMA Netw Open. 2021 Mar 29;4(3):e213497. doi: 10.1001/jamanetworkopen.2021.3497 (PMC8008287; doi:10.1001/jamanetworkopen.2021.3497)
Supplement: Supplement. — eAppendix. Patient Level Simulation Model eFigure 1. Forest Plot of NMA Results NTE MM for PFS Line 1 eFigure 2. Forest Plot of NMA Results R/R MM for PFS Line 2 eFigure 3. Forest Plot of NMA Results R/R MM for PFS Line 3 eTable 1. Treatment Sequences Including Relative Effectiveness as Obtained From NMA eTable 2. Details of the Treatment Regimens eTable 3. Unit Costs of Resource Use eTable 4. Costs of Additional Resource Use per Month eTable 5. Drug Costs per Regimen per Day and Month Stratified by Treatment Period eTable 6. Detailed Discounted and Undiscounted Effects and Costs for Each Treatment Sequence [file jamanetwopen-e213497-s001.pdf]

Supplemental Online Content

Blommestein HM, Franken MG, van Beurden-Tan CHY, et al. Cost-effectiveness of novel treatment sequences for transplant-ineligible patients with multiple myeloma. *JAMA Netw Open*. 2021;4(3):e213497. doi:10.1001/jamanetworkopen.2021.3497

eAppendix. Patient Level Simulation Model

eFigure 1. Forest Plot of NMA Results NTE MM for PFS Line 1

eFigure 2. Forest Plot of NMA Results R/R MM for PFS Line 2

eFigure 3. Forest Plot of NMA Results R/R MM for PFS Line 3

eTable 1. Treatment Sequences Including Relative Effectiveness as Obtained From NMA

eTable 2. Details of the Treatment Regimens

eTable 3. Unit Costs of Resource Use

eTable 4. Costs of Additional Resource Use per Month

eTable 5. Drug Costs per Regimen per Day and Month Stratified by Treatment Period

eTable 6. Detailed Discounted and Undiscounted Effects and Costs for Each Treatment Sequence

This supplemental material has been provided by the authors to give readers additional information about their work.

eAppendix. Patient Level Simulation Model

Regression coefficients to model time-to-event for line 1

Weibull model					
**Variable**	**Coefficient**	**SE**	**p-value**	**[95% Conf.**	**Interval]**
Constant	4.206	0.450	0.000	3.323	5.089
Age1	-0.022	0.005	0.000	-0.032	-0.012
Sex (Reference category male)	0.195	0.068	0.004	0.062	0.328
WHO status 0	Reference category				
WHO status 1	-0.112	0.082	0.170	-0.273	0.048
WHO status 2	-0.349	0.118	0.004	-0.583	-0.114
WHO status 3	-0.853	0.210	0.000	-1.273	-0.433
WHO status 4	-1.492	0.482	0.005	-2.488	-0.496
Haemoglobin1	0.115	0.028	0.000	0.059	0.171
Serum calcium1	-0.201	0.099	0.043	-0.395	-0.006
Melphalan-Thalidomide	Reference category				
Melphalan-Prednisone	-0.321	0.080	0.000	-0.478	-0.165
Treatment1 Bortezomib combination	-0.022	0.145	0.880	-0.307	0.263
Treatment1 Lenalidomide combination	0.293	0.178	0.100	-0.056	0.642
Treatment1 Other combination	-0.664	0.128	0.000	-0.915	-0.414
Comorbidity (Reference category no comorbidity)	-0.265	0.091	0.004	-0.446	-0.085
Shape	1.058	0.029		1.003	1.116

Regression coefficients to model type of event line 1

**Variable**	**Coefficient**	**SE**	**p-value**	**[95% Conf.**	**Interval]**
Constant	-6.777	1.068	0.000	-10.118	-5.930
TTE1 (ln)	-0.850	0.078	0.000	-0.848	-0.541
Age event1	0.109	0.014	0.000	0.089	0.142

Regression coefficients to model time-to-event for line 2

Weibull model					
Constant	2.517	0.086	29.340	0.000	2.349
Treatment BorDex	Reference category				
Treatment no BorDex	0.201	0.103	1.940	0.052	-0.002
Shape	1.025	0.039	0.000	0.950	1.105

Regression coefficients to model type of event line 2

**Variable**	**Coefficient**	**SE**	**p-value**	**[95% Conf.**	**Interval]**
Constant	-2.687	1.372	-1.960	-5.376	0.002
TTE2 (ln)	-0.727	0.109	-6.640	-0.942	-0.513
Age2	0.051	0.018	2.770	0.015	0.087

Regression coefficients to model time-to-event (death) for line 3

© 2021 Blommestein HM et al. *JAMA Network Open.*

Weibull model

Variable	Coefficient	SE	p-value	[95% Conf.	Interval]
Constant	2.543	0.728	0.414	-0.836	2.026
Treatment LenDex	Reference category				
Treatment no LenDex	-0.316	0.131	-2.420	0.016	-0.573
Shape	1.033	0.067		0.909	1.174

© 2021 Blommestein HM et al. *JAMA Network Open.*

eFigure 1. Forest Plot of NMA Results NTE MM for PFS Line 1

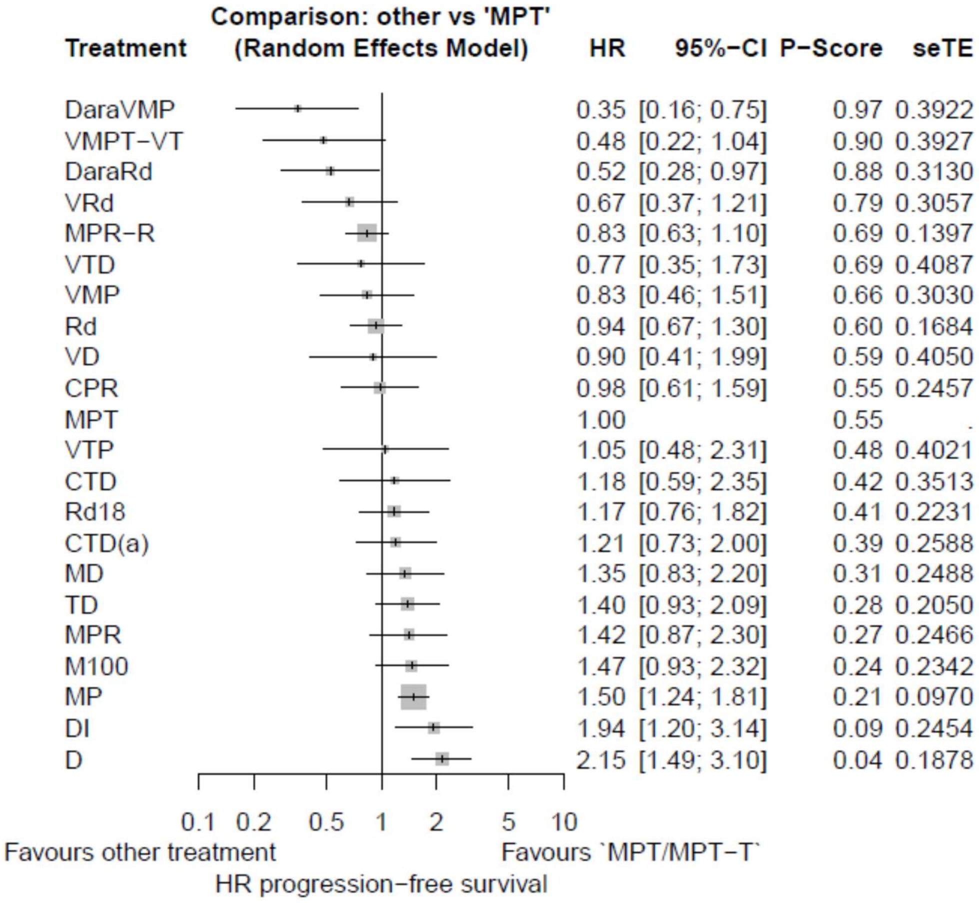

Reference: Blommestein et al. Haeamtologica 2019. Appendix 6, supplemental material and update Facon 2019[100].

D: Dexamethasone
DI: Dexamethasone-Interferon alpha
M100: Melphalan 100
MD: Melphalan-Dexamethasone
MP:Melphalan-Prednisone
TD: Thalidomide-Dexamethasone
CTD: Cyclophosphamide-Thalidomide-Dexamethasone
CTD(a): Cyclophosphamide-Thalidomide-Dexamethasone (attenuated)
MPT/MPT-T: Melphalan-Prednisone-Thalidomide / Melphalan-Prednisone-Thalidomide and Thalidomide maintenance
VD: Bortezomib-Dexamethasone
VTD: Bortezomib-Thalidomide-Dexamethasone
VMP: Bortezomib-Melphalan-Prednisone
VTP: Bortezomib-Thalidomide-Prednisone
VMPT-VT: Bortezomib-Melphalan-Prednisone-Thalidomide and Bortezomib-Thalidomide
CPR: Cyclophosphamide-Prednisone-Lenalidomide
Rd: Lenalidomide-Dexamethasone
Rd18: 18 cycles Lenalidomide-Dexamethasone
MPR: Melphalan-Prednisone-Lenalidomide
MPR-R: Melphalan-Prednisone-Lenalidomide and Lenalidomide maintenance
VRd: Bortezomib-Lenalidomide-Dexamethasone
DaraVMP: Daratumumab-Bortezomib-Melphalan-Prednisone

© 2021 Blommestein HM et al. *JAMA Network Open.*

eFigure 2. Forest Plot of NMA Results R/R MM for PFS Line 2

Treatment	% being best treatment	Hazard Ratio vs BorDex 95% CrI, PFS
DaraLenDex	99%	0.2 [0.13,0.3]
CarLenDex	0%	0.37 [0.25,0.52]
EloLenDex	0%	0.37 [0.26,0.53]
DaraBorDex	1%	0.4 [0.28,0.54]
IxaLenDex	0%	0.4 [0.27,0.57]
CarDex	0%	0.53 [0.44,0.64]
LenDex	0%	0.53 [0.39,0.71]
PeglDoxBor	0%	0.55 [0.43,0.71]
PanoBorDex	0%	0.63 [0.52,0.76]
BorThalDex	0%	0.7 [0.48,1]
PomDex	0%	0.73 [0.53,0.98]
VorinoBor	0%	0.77 [0.63,0.93]
Bor/BorDex	0%	1
Thal/ThalDex	0%	1.14 [0.9,1.42]
Dex	0%	1.49 [1.2,1.88]
OblDex	0%	1.63 [1.1,2.33]

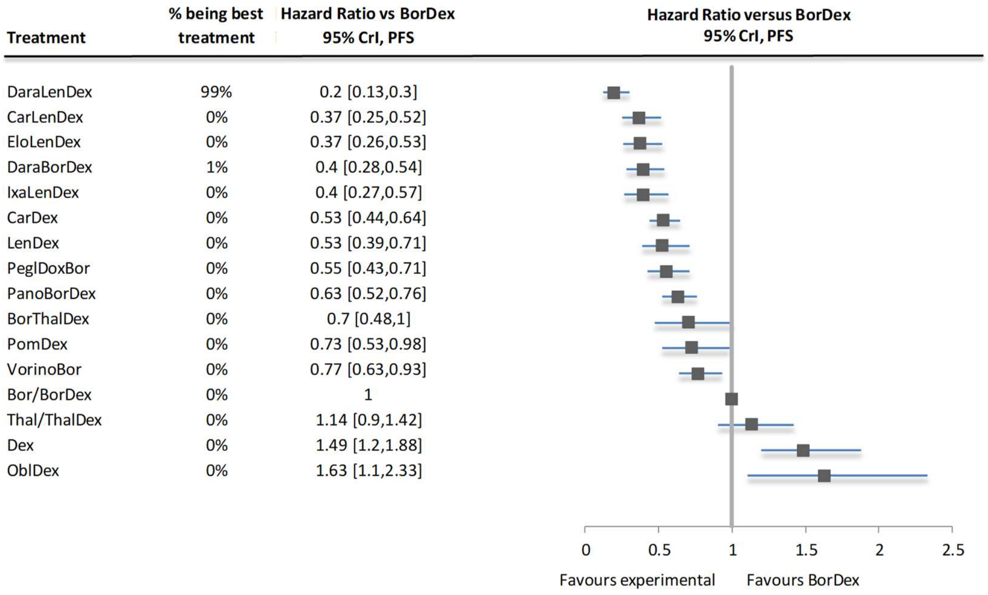

Reference: van Beurden et al. JCO 2017. Pomalidomide, bortezomib, dexamethasone (PomBorDex) added to the NMA.

© 2021 Blommestein HM et al. *JAMA Network Open.*

eFigure 3. Forest Plot of NMA Results R/R MM for PFS Line 3

Treatment	% being best treatment	Hazard Ratio vs LenDex 95% CrI, PFS
DaraLenDex	99%	0.37 [0.27,0.51]
CarLenDex	0%	0.69 [0.57,0.83]
EloLenDex	0%	0.7 [0.57,0.85]
DaraBorDex	1%	0.76 [0.48,1.15]
IxaLenDex	0%	0.75 [0.59,0.94]
CarDex	0%	1.03 [0.71,1.44]
LenDex	0%	1
PeglDoxBor	0%	1.07 [0.71,1.55]
PanoBorDex	0%	1.22 [0.84,1.71]
BorThalDex	0%	1.35 [0.89,1.95]
PomDex	0%	1.39 [1.02,1.84]
VorinoBor	0%	1.49 [1.03,2.09]
Bor/BorDex	0%	1.89 [1.41,2.56]
Thal/ThalDex	0%	2.18 [1.67,2.79]
Dex	0%	2.84 [2.34,3.47]
OblDex	0%	3.11 [2.13,4.38]

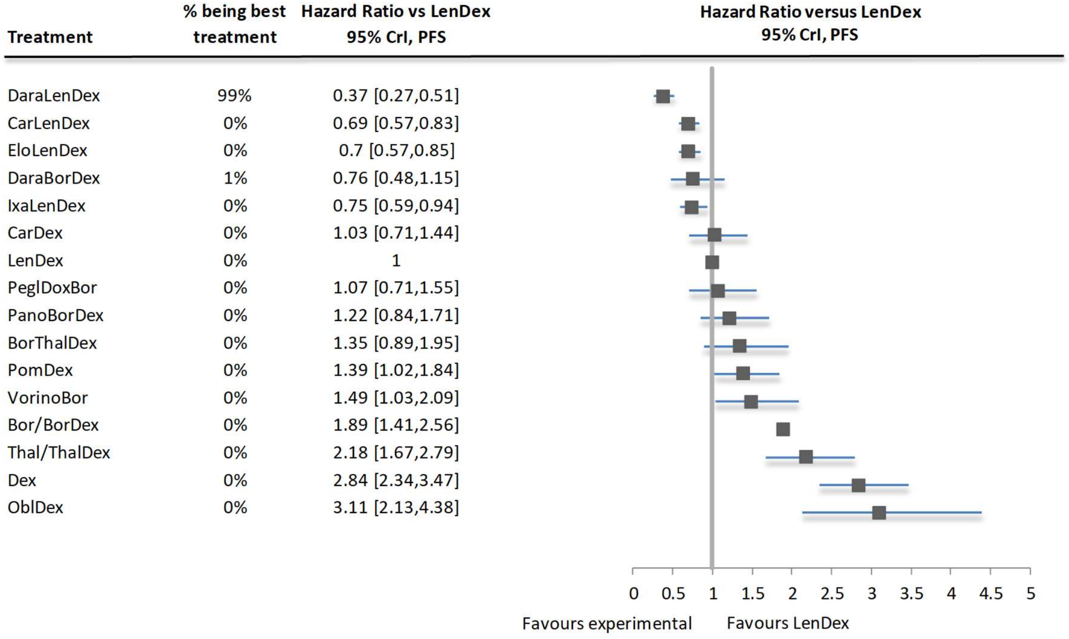

Reference: van Beurden et al. JCO 2017. Pomalidomide, bortezomib, dexamethasone (PomBorDex) added to the NMA.

© 2021 Blommestein HM et al. *JAMA Network Open.*

eTable 1. Treatment Sequences Including Relative Effectiveness as Obtained From NMA

Sequence			HR [95%CI] from NMA compared to reference case		
**Line 1**	**Line 2**	**Line 3**			
MPT	BorDex	LenDex			
VMP	LenDex	CarDex	VMP vs. MPT: 0.83 [0.46-1.51]	LenDex vs. BorDex: 0.53 [0.39-0.71]	CarDex vs. LenDex: 1.03 [0.71-1.44]
VMP	LenDex	PomDex	VMP vs. MPT: 0.83 [0.46-1.51]	LenDex vs. BorDex: 0.53 [0.39-0.71]	PomDex vs. LenDex: 1.39 [1.02-1.84]
VMP	CarLenDex	DaraBorDex	VMP vs. MPT: 0.83 [0.46-1.51]	CarLenDex vs. BorDex: 0.37 [0.25-0.52]	DaraBorDex vs. LenDex: 0.76 [0.48-1.15]
VMP	CarLenDex	PomDex	VMP vs. MPT: 0.83 [0.46-1.51]	CarLenDex vs. BorDex: 0.37 [0.25-0.52]	PomDex vs. LenDex: 1.39 [1.02-1.84]
VMP	DaraLenDex	CarDex	VMP vs. MPT: 0.83 [0.46-1.51]	DaraLenDex vs. BorDex: 0.20 [0.13-0.30]	CarDex vs. LenDex: 1.03 [0.71-1.44]
VMP	DaraLenDex	PomDex	VMP vs. MPT: 0.83 [0.46-1.51]	DaraLenDex vs. BorDex: 0.20 [0.13-0.30]	PomDex vs. LenDex: 1.39 [1.02-1.84]
VMP	DaraLenDex	PomBorDex	VMP vs. MPT: 0.83 [0.46-1.51]	DaraLenDex vs. BorDex: 0.20 [0.13-0.30]	PomBorDex vs. LenDex: 1.19 [0.80-1.70]
LenDex	CarDex	DaraBorDex	LenDex vs. MPT: 0.94 [0.67-1.30]	CarDex vs. BorDex: 0.53 [0.44-0.64]	DaraBorDex vs. LenDex: 0.76 [0.48-1.15]
LenDex	CarDex	PomDex	LenDex vs. MPT: 0.94 [0.67-1.30]	CarDex vs. BorDex: 0.53 [0.44-0.64]	PomDex vs. LenDex: 1.39 [1.02-1.84]
LenDex	DaraBorDex	CarDex	LenDex vs. MPT: 0.94 [0.67-1.30]	DaraBorDex vs. BorDex: 0.40 [0.28-0.54]	CarDex vs. LenDex: 1.03 [0.71-1.44]
LenDex	DaraBorDex	PomDex	LenDex vs. MPT: 0.94 [0.67-1.30]	DaraBorDex vs. BorDex: 0.40 [0.28-0.54]	PomDex vs. LenDex: 1.39 [1.02-1.84]
MPVT-VT	CarDex	DaraLenDex	MPVT-VT vs. MPT: 0.48 [0.22-1.04]	CarDex vs. BorDex: 0.53 [0.44-0.64]	DaraLenDex vs. LenDex: 0.37 [0.27-0.51]
MPVT-VT	CarDex	PanoBorDex	MPVT-VT vs. MPT: 0.48 [0.22-1.04]	CarDex vs. BorDex: 0.53 [0.44-0.64]	PomBorDex vs. LenDex: 1.19 [0.80-1.70]
MPVT-VT	CarDex	PomDex	MPVT-VT vs. MPT: 0.48 [0.22-1.04]	CarDex vs. BorDex: 0.53 [0.44-0.64]	PomDex vs. LenDex: 1.39 [1.02-1.84]
MPVT-VT	CarLenDex	PanoBorDex	MPVT-VT vs. MPT: 0.48 [0.22-1.04]	CarLenDex vs. BorDex: 0.37 [0.25-0.52]	PanoBorDex vs. LenDex: 1.22 [0.84-1.71]
MPVT-VT	CarLenDex	PomDex	MPVT-VT vs. MPT: 0.48 [0.22-1.04]	CarLenDex vs. BorDex: 0.37 [0.25-0.52]	PomDex vs. LenDex: 1.39 [1.02-1.84]
MPVT-VT	CarLenDex	PomBorDex	MPVT-VT vs. MPT: 0.48 [0.22-1.04]	CarLenDex vs. BorDex: 0.37 [0.25-0.52]	PomBorDex vs. LenDex: 1.19 [0.80-1.70]
MPVT-VT	DaraLenDex	CarDex	MPVT-VT vs. MPT: 0.48 [0.22-1.04]	DaraLenDex vs. BorDex: 0.20 [0.13-0.30]	CarDex vs. LenDex: 1.03 [0.71-1.44]
MPVT-VT	DaraLenDex	PanoBorDex	MPVT-VT vs. MPT: 0.48 [0.22-1.04]	DaraLenDex vs. BorDex: 0.20 [0.13-0.30]	PanoBorDex vs. LenDex: 1.22 [0.84-1.71]
MPVT-VT	DaraLenDex	PomDex	MPVT-VT vs. MPT: 0.48 [0.22-1.04]	DaraLenDex vs. BorDex: 0.20 [0.13-0.30]	PomDex vs. LenDex: 1.39 [1.02-1.84]
MPVT-VT	DaraLenDex	PomBorDex	MPVT-VT vs. MPT: 0.48 [0.22-1.04]	DaraLenDex vs. BorDex: 0.20 [0.13-0.30]	PomBorDex vs. LenDex: 1.19 [0.80-1.70]
Dara-VMP	CarLenDex	PanoBorDex	Dara-VMP vs. MPT: 0.35 [0.16-0.75]	CarLenDex vs. BorDex: 0.37 [0.25-0.52]	PanoBorDex vs. LenDex: 1.22 [0.84-1.71]
Dara-VMP	CarLenDex	PomDex	Dara-VMP vs. MPT: 0.35 [0.16-0.75]	CarLenDex vs. BorDex: 0.37 [0.25-0.52]	PomDex vs. LenDex: 1.39 [1.02-1.84]
Dara-VMP	CarLenDex	PomBorDex	Dara-VMP vs. MPT: 0.35 [0.16-0.75]	CarLenDex vs. BorDex: 0.37 [0.25-0.52]	PomBorDex vs. LenDex: 1.19 [0.80-1.70]
Dara-VMP	EloLenDex	CarDex	Dara-VMP vs. MPT: 0.35 [0.16-0.75]	EloLenDex vs. BorDex: 0.37 [0.26-0.53]	CarDex vs. LenDex: 1.03 [0.71-1.44]
Dara-VMP	EloLenDex	PomDex	Dara-VMP vs. MPT: 0.35 [0.16-0.75]	EloLenDex vs. BorDex: 0.37 [0.26-0.53]	PomDex vs. LenDex: 1.39 [1.02-1.84]
Dara-VMP	EloLenDex	PanoBorDex	Dara-VMP vs. MPT: 0.35 [0.16-0.75]	EloLenDex vs. BorDex: 0.37 [0.26-0.53]	PanoBorDex vs. LenDex: 1.22 [0.84-1.71]
Dara-VMP	EloLenDex	PomBorDex	Dara-VMP vs. MPT: 0.35 [0.16-0.75]	EloLenDex vs. BorDex: 0.37 [0.26-0.53]	PomBorDex vs. LenDex: 1.19 [0.80-1.70]
VRD	CarDex	PomDex	VRd vs. MPT: 0.67 [0.37-1.21]	CarDex vs. BorDex: 0.53 [0.44-0.64]	PomDex vs. LenDex: 1.39 [1.02-1.84]
DaraRd	CarDex	PomDex	DaraRd vs. MPT: 0.52 [0.28-0.97]	CarDex vs. BorDex: 0.53 [0.44-0.64]	PomDex vs. LenDex: 1.39 [1.02-1.84]
DaraRd	CarDex	PomBorDex	DaraRd vs. MPT: 0.52 [0.28-0.97]	CarDex vs. BorDex: 0.53 [0.44-0.64]	PomBorDex vs. LenDex: 1.19 [0.80-1.70]

© 2021 Blommestein HM et al. *JAMA Network Open.*

eTable 2. Details of the Treatment Regimens

Treatment regimen	Start (days)	Stop (days)	Route	Dosage per administration		Dosage (incl waste)		Number of administrations in cycle	Length of cycle (days)	Number of cycles	Source
**MPV**											
**Period I**	**1**	**315**									
Bortezomib			s.c.	1.3	mg/m2	3.5	mg	4	35	9	Palumbo 2014
Melphalan			oral	9	mg/m2	18	mg	4	35	9	Palumbo 2014
Prednisone			oral	60	mg/m2	115	mg	4	35	9	Palumbo 2014
**VMPT-VT**											
**Period I**	**1**	**315**									
Bortezomib			s.c.	1.3	mg/m2	3.5	mg	4	35	9	Palumbo 2014
Melphalan			oral	9	mg/m2	18	mg	4	35	9	Palumbo 2014
Prednisone			oral	60	mg/m2	115	mg	4	35	9	Palumbo 2014
Thalidomide			oral	50	mg	50	mg	35	35	9	Palumbo 2014
**Period II**	**316**	**1046**									
Bortezomib			s.c.	1.3	mg/m2	3.5	mg	1	14	52	Palumbo 2014
Thalidomide			oral	50	mg	50	mg	14	14	52	Palumbo 2014
**Dara-VMP**											
**Period I**	**1**	**42**									
Bortezomib			s.c.	1.3	mg/m2	3.5	mg	8	42	1	Mateos 2017
Melphalan			oral	9	mg/m2	2	mg	4	42	1	Mateos 2017
Prednisone			oral	60	mg/m2	5	mg	4	42	1	Mateos 2017
Daratumumab			iv	16	mg/kg	400	mg	6	42	1	Mateos 2017
Dexamethasone			oral	20	mg	20	mg	6	42	1	Mateos 2017
**Period II**	**43**	**379**									
Bortezomib			s.c.	1.3	mg/m2	3.5	mg	4	42	8	Mateos 2017
Melphalan			oral	9	mg/m2	2	mg	4	42	8	Mateos 2017
Prednisone			oral	60	mg/m2	5	mg	4	42	8	Mateos 2017
Daratumumab			iv	16	mg/kg	400	mg	2	42	8	Mateos 2017
Dexamethasone			oral	20	mg	20	mg	2	42	8	Mateos 2017
**Period III**	**380**	**until progression**									
Daratumumab			iv	16	mg/kg	400	mg	1	28		Mateos 2017
Dexamethasone			oral	20	mg	20	mg	1	28		Mateos 2017
**LenDex**											
**Period I**	**1**	**until progression**									
Lenalidomide			oral	25	mg	25	mg	21	28		HOVON guidelines
Dexamethasone			oral	40	mg	40	mg	4	28		HOVON guidelines
**VRd**											
**Period I**	**1**	**168**									
Bortezomib			sub	1.3	mg/m2	3.5	mg	4	21	8	
Lenalidomide			oral	25	mg	25	mg	14	21	8	
Dexamethasone			oral	20	mg	20	mg	8	21	8	
**Period II**	**169**	**until progression**									
Lenalidomide			oral	25	mg	25	mg	21	28		

© 2021 Blommestein HM et al. *JAMA Network Open.*

Dexamethasone			oral	40	mg	40	mg	4	28		
**BorDex**											
**Period I**	**1**	**168**									
Bortezomib			s.c.	1.3	mg/m2	3.5	mg	4	21	8	HOVON guidelines
Dexamethasone			oral	20	mg	20	mg	8	21	8	HOVON guidelines
**Period II**	**169**	**274**									
Bortezomib			s.c.	1.3	mg/m2	3.5	mg	4	35	3	HOVON guidelines
Dexamethasone			oral	20	mg	20	mg	8	35	3	HOVON guidelines
**CarDex**											
**Period I**	**1**	**28**									
Carfilzomib			iv	56	mg/m2	100	mg	6	28	1	ENDEAVOR
Dexamethasone			oral	20	mg	20	mg	8	28	1	ENDEAVOR
**Period II**	**29**	**until progression**									
Carfilzomib			iv	56	mg/m2	1000	mg	6	28		ENDEAVOR
Dexamethasone			oral	40	mg	40	mg	8	28		ENDEAVOR
**CarLenDex**											
**Period I**	**1**	**336**									
Carfilzomib			iv	20 - 27	mg/m2	60	mg	6	28	12	Stewart 2015
Lenalidomide			oral	25	mg	25	mg	21	28	12	Stewart 2015
Dexamethasone			oral	40	mg	40	mg	4	28	12	Stewart 2015
**Period II**	**337**	**504**									
Carfilzomib			iv	27	mg/m2	60	mg	4	28	6	Stewart 2015
Lenalidomide			oral	25	mg	25	mg	21	28	6	Stewart 2015
Dexamethasone			oral	40	mg	40	mg	4	28	6	Stewart 2015
**Period III**	**505**	**until progression**									
Lenalidomide			oral	25	mg	25	mg	21	28		Stewart 2015
Dexamethasone			oral	40	mg	40	mg	4	28		Stewart 2015
**DaraBorDex**											
**Period I**	**1**	**63**									
Daratumumab			iv	16	mg/kg	1200		3	21	3	CASTOR
Bortezomib			sub	1.3	mg/m2	3.5		4	21	3	CASTOR
Dexamethasone			oral	20	mg	20		8	21	3	CASTOR
**Period II**	**64**	**169**									
Daratumumab			iv	16	mg/kg	1200		1	21	5	CASTOR
Bortezomib			sub	1.3	mg/m2	3.5		4	21	5	CASTOR
Dexamethasone			oral	20	mg	20		8	21	5	CASTOR
**Period III**	**170**	**until progression**									
Daratumumab			iv	16	mg/kg	1200		1	28		CASTOR
Dexamethasone			oral	20	mg	20		8	28		CASTOR
**DaraLenDex**											
**Period I**	**1**	**56**									
Daratumumab			iv	16	mg/kg	1200	mg	4	28	2	Pollux
Lenalidomide			oral	25	mg	25	mg	21	28	2	Pollux
Dexamethasone			oral	40	mg	40	mg	4	28	2	Pollux
**Period II**	**57**	**169**									
Daratumumab			iv	16	mg/kg	1200	mg	2	28	4	Pollux
Lenalidomide			oral	25	mg	25	mg	21	28	4	Pollux

© 2021 Blommestein HM et al. *JAMA Network Open.*

Dexamethasone			oral	40	mg	40	mg	4	28	4	Pollux
**Period III**	**170**	**until progression**									
Daratumumab			iv	16	mg/kg	1200	mg	1	28		Pollux
Lenalidomide			oral	25	mg	25	mg	21	28		Pollux
Dexamethasone			oral	40	mg	40	mg	4	28		Pollux
**EloLenDex**											
**Period I**	**1**	**56**									
Elotuzumab			iv	10	mg/kg	800	mg	4	28	2	Lional
Lenalidomide			oral	25	mg	25	mg	21	28	2	Lional
Dexamethasone			oral	40	mg	40	mg	4	28	2	Lional
**Period II**	**57**	**until progression**									
Elotuzumab			iv	10	mg/kg	800	mg	2	28		Lional
Lenalidomide			oral	25	mg	25	mg	21	28		Lional
Dexamethasone			oral	40	mg	40	mg	4	28		Lional
**PanoBorDex**											
**Period I**	**1**	**168**									
Panobinostat			oral	20	mg	20	mg	6	21	8	San Miguel
Bortezomib			sub	1.3	mg/m2	3.5	mg	4	21	8	San Miguel
Dexamethasone			oral	20	mg	20	mg	8	21	8	San Miguel
**Period II**	**169**	**337**									
Panobinostat			oral	20	mg	20	mg	12	42	4	San Miguel
Bortezomib			sub	1.3	mg/m2	3.5	mg	4	42	4	San Miguel
Dexamethasone			oral	20	mg	20	mg	8	42	4	San Miguel
**PomDex**											
**Period I**	**1**	**until progression**									
Pomadolidomide			oral	4	mg	4	mg	21	28		San Miguel 2013
Dexamethasone			oral	40	mg	40	mg	4	28		San Miguel 2013
**PomBorDex**											
**Period I**	**1**	**168**									
Pomalidomide			oral	4	mg	4	mg	14	21	8	Richardson 2018
Bortezomib			s.c.	1.3	mg/m2	3.5	mg	4	21	8	Richardson 2018
Dexamethason			oral	20	mg	20	mg	8	21	8	Richardson 2018
**Period II**	**169**	**until progression**									
Pomalidomide			oral	4	mg	4	mg	14	21	8	Richardson 2018
Bortezomib			s.c.	1.3	mg/m2	3.5	mg	2	21	8	Richardson 2018
Dexamethason			oral	20	mg	20	mg	4	21	8	Richardson 2018

© 2021 Blommestein HM et al. *JAMA Network Open.*

eTable 3. Unit Costs of Resource Use

Type of resource	Unit price	Unit	Source
**Hospital resource use**			
Inpatient day intensive care (incl. diagnostics and medication)	$2,546.00	per day	Dutch Costing Manual 2015, updated to dec 2019
Inpatient day haemato-oncology ward	$803.60	per day	Dutch Costing Manual 2015, updated to dec 2019
Day-care treatment*	$348.73	per visit	Dutch Costing Manual 2015, updated to dec 2019
Outpatient visit haemoto-oncology	$166.79	per visit	Dutch Costing Manual 2015, updated to dec 2019
Laboratory test (one unit added for each outpatient visit)	$73.64	per outpatient visit	Gaultney et al. 2013, updated to 2019
**Drugs**			
Bortezomib	$1,438.54	3.5 mg vial	medicijnkosten.nl, march 2019
Carfilzomib	$264.71	10 mg vial‡	medicijnkosten.nl, march 2019
Daratumumab	$523.80	100 mg vial	medicijnkosten.nl, march 2019
Daratumumab	$2,095.18	400 mg vial	medicijnkosten.nl, march 2019
Dexamethasone	$1.14	20 mg tablet	medicijnkosten.nl, march 2019
Dexamethasone	$1.55	40 mg capsula	medicijnkosten.nl, march 2019
Elotuzumab	$1,508.89	300 mg vial	medicijnkosten.nl, march 2019
Elotuzumab	$2,011.85	400 mg vial	medicijnkosten.nl, march 2019
Lenalidomide	$327.52	10 mg tablet	medicijnkosten.nl, march 2019
Lenalidomide	$361.47	25 mg tablet	medicijnkosten.nl, march 2019
Melphalan	$3.12	2 mg tablet	medicijnkosten.nl, march 2019
Panobinostat	$951.02	20 mg tablet	medicijnkosten.nl, march 2019
Pomalidomide	$602.67	4 mg capsula	medicijnkosten.nl, march 2019
Prednisone	$3.64	5 mg tablet	medicijnkosten.nl, march 2019
Thalidomide	$5.23	50 mg tablet	medicijnkosten.nl, march 2019
Thalidomide	$5.74	100 mg capsula	medicijnkosten.nl, march 2019

‡ Since mg price is similar for 10 mg, 30mg or 60 mg we worked with the price and volume of 10mg

© 2021 Blommestein HM et al. *JAMA Network Open.*

eTable 4. Costs of Additional Resource Use per Month

Type of resource	Use per month	Source	Reference price	Costs per month (Dollars, Euro converted(1=$1.2 143) 2019 price)
**Inpatient day intensive care**				
Line 1	0.07			$178.22
Line 2	0.02	PHAROS registry	$2,546	$50.92
Line 3	0.04			$101.84
**Inpatient day haemato-oncology**				
Line 1	1.7			$1,391.15
Line 2	1.5	PHAROS registry	$804	$1,188.85
Line 3	1.5			$1,240.89
**Outpatient visit haemato-oncology**				
Line 1	1.7			$399.58
Line 2	2.0	PHAROS registry	$240	$475.09
Line 3	2.2			$535.69
Note: price outpatient = 166+ 74 laboratory cost				

© 2021 Blommestein HM et al. *JAMA Network Open.*

eTable 5. Drug Costs per Regimen per Day and Month Stratified by Treatment Period

Treatment	Start period (day)	Stop period (day)	Costs per day	Costs per month (30.4 days)	Maximum duration (days)
**MPT**					
Total period	1	252	$29	$903	252
**VMP**					
Total period	1	315	$176	$5,364	315
**VMPT-VT**					
TOTAL period I	1	315	$182	$5,523	315
TOTAL period II	316	1046	$108	$3,282	730
**DaraVMP-Dara**					
Total Period I	1	42	$1,182	$35,936	42
Total Period II	64	169	$447	$13,570	336
Total Period III	170	progression	$251	$7,648	until progression**
**VRd**					
Total Period I	1	168	$515	$15,669	168
Total Period II	169	progression*	$271	$8,249	until progression**
**BorDex**					
Total period I	1	168	$274	$8,343	168
Total period II	169	274	$165	$5,005	105
**LenDex**					
Total Period	1	progression*	$271	$8,249	until progression**
**PomDex**					
Total Period	1	progression*	$452	$13,747	until progression**
**CarDex**					
Total Period I	1	28	$454	$13,805	28
Total Period II	29	progression*	$567	$17,258	until progression**
**CarLenDex**					
Total Period I	1	336	$555	$16,870	336
Total Period II	337	504	$460	$13,996	168
Total Period III	505	progression*	$271	$8,249	until progression**
**DaraLenDex**					
Total Period I	1	56	$1,169	$35,545	56
Total Period II	57	169	$720	$21,897	112
Total Period III	170	progression*	$495	$15,073	until progression**
**EloLenDex**					
Total Period I	1	56	$846	$25,723	56
Total Period II	57	progression*	$559	$16,986	until progression**
**PanoBorDex**					
Total Period I	1	168	$546	$16,603	168
Total Period II	169	337	$409	$12,432	168
**DaraBorDex**					
Total Period I	1	63	$1,172	$35,640	63
Total Period II	64	169	$573	$17,442	105

© 2021 Blommestein HM et al. *JAMA Network Open.*

Total Period III	170	progression*	$225	$6,834	until progression**
**PomBorDex**					
Total period I	1	168	$676	$20,557	168
Total period II	169	progression*	$539	$16,386	until progression**

MPT: melphalan-prednisone-thalidomide
VMP: melphalan-prednisone-bortezomib
VMPT-VT: melphalan-prednisone-bortezomib-thalidomide - bortezomib-thalidomide
DaraVMP-Dara: daratumumab-melphalan-prednisone-bortezomib – daratumumab
VRd: bortezomib-lenalidomide-dexamethasone
BorDex: bortezomib-dexamethasone
LenDex: lenalidomide-dexamethasone
PomDex: pomalidomide-dexamethasone
CarDex: carfilzomib-dexamethasone
CarLenDex: carfilzomib-lenalidomide-dexamethasone
DaraLenDex: daratumumab-lenalidomide-dexamethasone
EloLenDex: elotuzumab-lenalidomide-dexamethasone
PanoBorDex: panobinostat -lenalidomide-dexamethasone
DaraBorDex: daratumumab-bortezomib-dexamethasone
PomBorDex: pomalidomide-bortezomib-dexamethasone

© 2021 Blommestein HM et al. *JAMA Network Open.*

eTable 6. Detailed Discounted and Undiscounted Effects and Costs for Each Treatment Sequence

Line 1	Line 2	Line 3	OS months	OS Median	OS disc	QALYs	QALYs disc	Costs	Cost disc	EFS L1	EFS L2	EFS L3	TCOST L1	TCOST L2	TCOST L3	DCOST L1	DCOST L2	DCOST L3	N Line 1	N Line 2	N Line 3
VMP	LenDex	CarDex	51	41	49	3.2	3.0	369435	321034	32	23	12	119471	230251	252329	46685	190529	202905	100%	64%	41%
VMP	LenDex	PomDex	50	40	48	3.1	2.9	324854	285619	32	23	9	119073	229805	144700	46651	190190	127345	100%	64%	41%
VMP	CarLenDex	DaraBorDex	59	48	56	3.7	3.5	517138	441876	32	33	15	118987	459366	240232	46646	375441	193103	100%	64%	44%
VMP	CarLenDex	PomDex	57	45	54	3.5	3.3	475340	409243	31	33	9	118693	459645	144832	46621	375798	127421	100%	64%	44%
VMP	DaraLenDex	CarDex	75	58	69	4.6	4.3	927974	733277	32	59	12	119142	1081947	250771	46678	954208	201639	100%	64%	47%
VMP	DaraLenDex	PomDex	74	56	68	4.6	4.2	878891	697907	32	59	9	118871	1084534	144185	46630	956543	126864	100%	64%	47%
VMP	DaraLenDex	PomBorDex	74	57	69	4.6	4.3	918078	726470	32	59	11	119126	1084853	226812	46672	956953	191821	100%	64%	47%
LenDex	CarDex	DaraBorDex	48	39	46	3.0	2.8	682880	601285	28	23	15	281758	484281	239506	227747	392838	192582	100%	63%	40%
LenDex	CarDex	PomDex	46	36	44	2.8	2.7	643342	568884	28	23	9	281515	482922	144504	227539	391753	127180	100%	63%	40%
LenDex	DaraBorDex	CarDex	52	41	49	3.2	3.0	625403	549399	27	31	12	280404	378067	252329	226673	300591	202923	100%	63%	43%
LenDex	DaraBorDex	PomDex	51	40	48	3.1	3.0	581074	514529	28	31	9	281288	378465	144436	227374	300893	127081	100%	63%	43%
VMPT-VT	CarDex	DaraLenDex	80	67	74	4.9	4.6	776667	635948	53	23	27	228132	479923	538048	101094	389306	472966	100%	68%	42%
VMPT-VT	CarDex	PanoBorDex	73	59	68	4.5	4.2	612333	515869	53	23	10	228528	481494	139626	101145	390499	108473	100%	68%	42%
VMPT-VT	CarDex	PomDex	72	58	67	4.5	4.2	612021	515422	53	23	9	227325	480499	144099	100928	389736	126796	100%	68%	42%
VMPT-VT	DaraLenDex	CarDex	99	83	90	6.1	5.6	1069489	823321	53	58	12	228966	1065892	250447	101347	940212	201366	100%	68%	48%
VMPT-VT	DaraLenDex	PanoBorDex	98	81	89	6.0	5.5	1014305	786024	53	58	10	228069	1065171	138813	101106	939596	107948	100%	68%	48%
VMPT-VT	DaraLenDex	PomDex	97	81	89	6.0	5.5	1019350	788832	53	58	9	228704	1066973	143799	101217	941100	126524	100%	68%	48%
VMPT-VT	DaraLenDex	PomBorDex	98	82	89	6.1	5.5	1059735	816742	54	58	10	229251	1068070	225752	101353	942149	190949	100%	68%	48%
VMPT-VT	CarLenDex	PanoBorDex	80	66	74	4.9	4.6	598834	501931	53	33	10	227752	456557	139737	101020	373193	108551	100%	68%	45%
VMPT-VT	CarLenDex	PomDex	80	65	74	4.9	4.6	601757	503478	53	33	9	228480	456559	144255	101175	373133	126948	100%	68%	45%
VMPT-VT	CarLenDex	PomBorDex	80	66	74	5.0	4.6	638927	531163	53	33	11	228471	455892	228960	101150	372691	193624	100%	68%	45%
DaraVMP	CarLenDex	PanoBorDex	98	82	89	6.0	5.5	1180320	963281	71	33	10	808689	454328	139013	640177	371349	108070	100%	68%	44%
DaraVMP	CarLenDex	PomDex	98	81	89	6.0	5.5	1186605	967078	71	33	9	812943	453496	144160	643595	370762	126889	100%	68%	44%
DaraVMP	CarLenDex	PomBorDex	97	81	89	6.0	5.5	1215733	988834	70	33	11	803706	456090	226973	636554	372718	191991	100%	68%	44%
DaraVMP	EloLenDex	CarDex	97	82	89	6.0	5.5	1350621	1085794	70	32	12	803069	640812	249365	635894	560175	200531	100%	68%	44%
DaraVMP	EloLenDex	PanoBorDex	97	81	89	6.0	5.5	1311012	1057083	71	32	10	809713	644434	139266	640978	563322	108216	100%	68%	44%
DaraVMP	EloLenDex	PomDex	97	81	89	6.0	5.5	1311609	1057142	71	32	9	810800	639866	144282	641935	559316	126974	100%	68%	44%
DaraVMP	EloLenDex	PomBorDex	97	81	89	6.0	5.5	1345024	1081747	71	32	11	806970	641080	226916	638919	560404	191933	100%	68%	44%
VRD	CarDex	PomDex	58	47	55	3.6	3.4	828936	717756	39	23	9	451183	481353	144859	363560	390459	127461	100%	66%	42%
DaraLenDex	CarDex	PomBorDex	69	56	64	4.3	4.0	1336944	1139944	49	23	11	916625	481432	226690	798973	390552	191716	100%	68%	42%
DaraLenDex	CarDex	PomDex	68	55	64	4.2	3.9	1298004	1110003	49	23	9	912537	480894	144471	795228	390105	127153	100%	68%	42%

© 2021 Blommestein HM et al. *JAMA Network Open.*
